# A detailed analysis of body composition in relation to cardiopulmonary exercise test indices

**DOI:** 10.1038/s41598-024-72973-0

**Published:** 2024-09-16

**Authors:** Per Karlsson, Robin Strand, Joel Kullberg, Karl Michaëlsson, Håkan Ahlström, Lars Lind, Andrei Malinovschi

**Affiliations:** 1 Department of Medical Sciences: Clinical Physiology, Uppsala University, Uppsala, Sweden; 2 Division of Radiology, Department of Surgical Sciences, Uppsala University, Uppsala, Sweden; 3 Department of Information Technology, Centre for Image Analysis, Uppsala University, Uppsala, Sweden; 4 Division of Radiology, Department of Surgical Sciences, Uppsala University, Antaros Medical AB, BioVenture Hub, Uppsala, Mölndal, Sweden; 5 Department of Surgical Sciences, Unit of Medical Epidemiology, Uppsala University, Uppsala, Sweden; 6 Department of Medical Sciences, Cardiovascular Epidemiology, Uppsala University, Uppsala, Sweden

**Keywords:** Cardiopulmonary exercise test, Body composition, MRI, Obesity, Respiration, Magnetic resonance imaging

## Abstract

A cardiopulmonary exercise test (CPET) is a test assessing an individual’s physiological response during exercise. Results may be affected by body composition, which is best evaluated through imaging techniques like magnetic resonance imaging (MRI). The aim of this study was to assess relationships between body composition and indices obtained from CPET. A total of 234 participants (112 female), all aged 50 years, underwent CPETs and whole-body MRI scans (> 1 million voxels). Voxel-wise statistical analysis of tissue volume and fat content was carried out with a method called Imiomics and related to the CPET indices peak oxygen consumption (V̇O_2peak_), V̇O_2peak_ scaled by body weight (V̇O_2kg_) and by total lean mass (V̇O_2lean_), ventilatory efficiency (V̇E/V̇CO_2_-slope), work efficiency (**Δ**V̇O_2_/**Δ**WR) and peak exercise respiratory exchange ratio (RERpeak). V̇O_2peak_ showed the highest positive correlation with volume of skeletal muscle. V̇O_2kg_ negatively correlated with tissue volume in subcutaneous fat, particularly gluteal fat. RERpeak negatively correlated with tissue volume in skeletal muscle, subcutaneous fat, visceral fat and liver. Some associations differed between sexes: in females **Δ**V̇O_2_/**Δ**WR correlated positively with tissue volume of subcutaneous fat and V̇E/V̇CO_2_-slope with tissue volume of visceral fat, and, in males, V̇O_2peak_ correlated positively to lung volume. In conclusion, voxel-based Imiomics provided detailed insights into how CPET indices were related to the tissue volume and fat content of different body structures.

## Introduction

The cardiopulmonary exercise test (CPET) is used in clinical practice to analyse exchange of breathing gases, typically oxygen and carbon dioxide, during exercise^1,2^.

One of the principal outcomes obtained by CPET is the maximal amount of oxygen consumption (V̇O_2peak_), commonly scaled by body weight (V̇O2kg) or sometimes, when available, by lean mass (V̇O_2lean_). V̇O_2peak_ has been shown to be a strong predictor of cardiovascular disease and all-cause mortality^3–11^. Other common indices obtained from CPET, and also used in clinical practice, are ventilatory efficiency (V̇E/V̇CO_2_-slope), work efficiency (**Δ**V̇O_2_/**Δ**WR), peak exercise respiratory exchange ratio (RERpeak) and oxygen pulse (V̇O_2HR_).

Imiomics (imaging-omics) is a whole-body magnetic resonance imaging (MRI) concept by which it is possible to analyse, relative to other individuals, each voxel for local tissue volume or fat content. The voxels can thereby be correlated to non-imaging data, e.g. variables from CPET^6,7^. The results are presented as colour-coded anatomical images; displaying the strength of correlation between the tissue volume or fat content with the non-imaging data.

Excessive adipose tissue located around internal organs, also known as visceral fat, is a risk factor for cardiovascular diseases and type-2 diabetes mellitus (independent of total fat mass)^8–11^, and is also linked with diastolic dysfunction and lung function impairment^12–18^. Furthermore, it is possible that visceral fat possess different physiologic impact in different anatomical regions; for example, fat around the heart could directly affect the function of the heart muscle^11^.

Consequently, we hypothesize that volume of visceral fat is associated with the CPET indices (which are affected by heart and lung function); furthermore, we speculate that there is a different strength of this association depending upon the location of the fat.

Several of the previous studies that have examined the associations between CPET and body composition have focused on the appropriate way to scale V̇O_2peak_^19,22^. In general, they indicate that scaling V̇O2peak by body weight result in overcorrection in larger individuals whereas scaling by lean mass may be an alternative not as biased by size and body composition^19–22^.

However, in this study, the aim is to utilize the Imiomics technique to provide new knowledge, in great anatomical detail, about how the location of fat storage and distribution of body mass relates to the aforementioned CPET indices.

## Method

We selected a random sample of 50-year-olds living in Uppsala from the Swedish Population Register^23^. The invitation to our investigation was sent out the month after their 50th birthday. There were no exclusion criteria. A total of 502 individuals were enrolled, with a participation rate of 25%. None of the participants had performed an exercise test with gas exchange before. The Ethics Committee of Uppsala University approved the study (No. 2009/057), and the participants filled in an informed consent document. The research was conducted in accordance with the Declaration of Helsinki.

During the initial visit, which took place after an overnight fast, weight was measured by an electronic scale (Tanita BC-418MA, Tokyo, Japan) and height was measured by a measuring tape attached to a wall. The measurement error of the height and weight measurements were not investigated. BMI was calculated as weight divided by height squared (kg·m − 2). Waist circumference was measured at the level of the umbilicus. Lipids were measured at the Department of Clinical Chemistry, Uppsala University Hospital, by standard techniques. The participants answered a questionnaire about lifestyle, medical history, medication and smoking was used. To measure physical activity, two questions were used “How many times a week do you engage in light activity for 30 min?” and “How many times a week do you engage in hard exercise for 30 min?”. From these two questions, four groups were defined: Sedentary (light activity only, < 2 times a week), Light (light activity only, > 1 times a week), Moderate (hard exercise 1 or 2 times a week) and High (hard exercise > 2 times a week).

### Inclusion for analysis

Participants with a peak heart rate of less than 85% of predicted (predicted heart rate calculated as 220 minus age) and/or respiratory exchange ratio (RERpeak) of less than 1 were excluded from the analysis, as we aimed to include only those who underwent a maximal or near maximal test. In order to minimise the potential impact of smoking on ventilatory parameters, individuals who were actively smoking were excluded from the study. Furthermore, due to resource constraints, not all participants underwent MRI scans. A few participants were excluded due to low image quality of the MRI images. None of the participants were denied inclusion due to concurrent diseases.

### Cardiopulmonary exercise test (CPET)

Within 1 week from the first visit the participants returned non-fasted for the CPET.

The test was conducted by each participant on a stationary bicycle ergometer (Ergose-lect 100/200, Ergoline, Germany). The breath-by-breath technique was used to measure breathing gases (Jaeger Oxycon Pro, Erich Jaeger, Germany). The participants performed a ramp test with an incremental workload of 10 watts every minute. The initial workload was 50 watts for males and 30 watts for females. No warm-up was used as this was not implemented in the clinical protocol during this time. Participants were instructed to continue the test for as long as possible and not stop before complete fatigue. After the exercise stage the participants remained seated on the bike for 5 min.

The examinations took place during the afternoon at a laboratory next to Uppsala University hospital in a room with a constant temperature of 22 C.

The devices and protocols used for CPET followed the recommendations from ATS/ACCP^24^.

V̇E/V̇CO_2_-slope was calculated with regression and defined as the slope between ventilation (V̇E) and ventilation of carbon dioxide (V̇CO_2_) from the start of exercise to the respiratory compensation point (if achieved). **Δ**VO_2_/**Δ**WR was defined as the slope between ventilation of oxygen (V̇O_2_) and work rate (WR) from the start of work to maximal exercise, calculated with regression. The regressions were performed by the software included in the Jaeger equipment. To get V̇O_2HR_ we divided V̇O_2peak_ by peak heart rate obtained by electrocardiogram (Jaeger Oxycon Pro, Erich Jaeger, Germany). V̇O_2lean_ was calculated by dividing V̇O_2peak_ by lean mass, acquired through DXA.

### Dual-energy X-ray absorptiometry (DXA)

During the initial visit a dual-energy X-ray absorptiometry scanner (DXA; Lunar Prodigy, GE Healthcare, USA) was used to measure fat and lean mass. The examinations were all performed by the same nurse. The precision error for total fat was 1.5% and that for lean mass was 1.0%. The error was calculated using the International Society for Clinical Densitometry recommendations^25^.

Automatic edge detection was used. However, all scans were checked for errors and, if needed, manually corrected. The fat mass measured with the narrow fan beam DXA equipment is 1.7–2% higher than that measured by a four-compartment model^26^.

### Magnetic resonance imaging (MRI)

MRI was performed on participants in the supine position with a 1.5T clinical MRI system (Philips Achieva, Philips Healthcare, Best, Netherlands). We employed a whole-body water-fat imaging protocol utilizing a spoiled 3D multi-gradient echo sequence. The MRI scanning results in images showing quantitative water and fat fractions, from which the fat percentage signal value can be measured in all voxels in the body. The scan parameters were set as follows: repetition time (TR)/echo time 1 (TE1)/echo spacing (ΔTE) = 5.9/1.36/1.87 ms, with acquisition of 3 unipolar echoes and a flip angle of 3 degrees. The imaged field of view (FOV) measured 530 × 377 × 2000 mm^3^, with reconstructed voxel dimensions of 2.07 × 2.07 × 8.0 mm^3^ in the left-right, anterior-posterior, and foot-head directions, respectively. Imaging and image reconstruction methodology has been described in greater detail previously^6,27^. The MRI was done within one month from the first visit.

### Imiomics

The so called Imiomics method uses image registrations to deform all 3D image data into a common geometry. This allows point-to-point (voxel-wise) comparisons of the 3D image data between all subjects. By analysing the local volume changes needed for the image registration between subjects, correlations with tissue volume in the analysed cohort can be studied. Similarly, by comparing the voxel-wise image intensities (here, quantitative measures of voxel-wise fat content) one can analyse correlation to tissue fat content throughout the body. More details on the Imiomics methodology can be found elsewhere^6,27^.

### Statistics

For descriptive statistics, R version 4.2.2. was used. Table 1 was created with tableone package^28^.

**Table 1:** Characteristics of participants included for analysis. Data are presented as mean (SD) unless otherwise indicated.

	Male	Female
n	122	112
Height, cm	179.5 (6.5)	166.8 (6.4)
Weight, kg	85.0 (11.3)	70.7 (12.4)
Waist circumference, cm	93.5 (9.5)	88.5 (11.3)
BMI, kg/m^2^	26.4 (3.4)	25.5 (4.5)
V̇O_2peak_, mL*min^-1^	2878.1 (516.5)	1911.9 (359.4)
V̇O_2kg_, mL*kg^--1^*min^-1^	34.3 (7.2)	27.6 (6.0)
V̇O_2lean_, mL^*^kg^--1^*min^-1^	48.1 (6.9)	45.9 (7.7)
V̇O_2HR_	17.0 (3.0)	11.4 (2.2)
VE/VCO_2_-slope	27.1 (3.4)	25.5 (4.0)
RERpeak	1.1 (0.1)	1.1 (0.1)
ΔV̇O_2_/ΔWR	11.2 (1.2)	10.2 (1.1)
Peak workload, watt	230.7 (41.5)	161.1 (32.1)
Physical activity group		
Sedentary	19 (15.8)	8 (7.2)
Light	12 (10.0)	23 (20.7)
Moderate	48 (40.0)	46 (41.4)
High	41 (34.2)	34 (30.6)
Diabetes mellitus	1 (0.8)	2 (1.8)
Angina pectoris	0 (0)	0 (0)
Anti hypertensive treatment	6 (4.9)	6 (5.4)
Total cholesterol, mmmol*L^-1^	5.4 (1.0)	5.2 (0.9)
LDL-cholesterol, mmol*L^-1^	3.5 (0.9)	3.2 (0.8)

BMI: body mass index, V̇O_2peak_: peak oxygen consumption, V̇O_2kg_: peak oxygen consumption divided by body weight, V̇O_2lean_: peak oxygen consumption divided by lean mass, V̇O_2HR_: peak oxygen consumption divided by peak heart rate, V̇E/V̇CO_2_ slope: ventilatory efficiency (slope between ventilation and ventilation of carbon dioxide), RERpeak: respiratory exchange ratio at peak exercise, ΔV̇O_2_/ΔWR: work efficiency (slope between ventilation of oxygen and work rate), LDL: low-density lipoprotein.

The CPET measurements were visually inspected by histograms and density curves and interpreted as normally distributed. Pearson’s correlations were used to assess the relationship between CPET variables and voxels on MRI.

The results are shown in images where voxels with a significant (*p* < 0.05) correlation coefficient are coloured. Red represents a strong positive correlation and blue represents a strong negative correlation. The figures were interpreted visually. Only significant correlations in many adjacent voxels, in the same anatomical structure, were reported in the text as a finding.

## Results

**Fig. 1:**
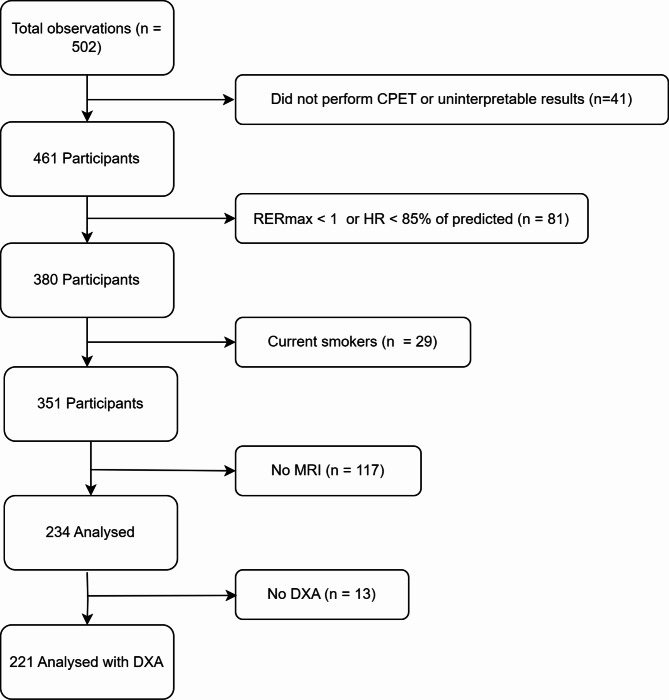
Flowchart of the process for including and excluding participants for final analysis.

A total of 234 participants were included for analysis. Of these, 13 did not undergo DXA; therefore, 221 were analysed regarding V̇O_2lean_. Figure 1 shows a flowchart of the exclusion process.

The participant characteristics are shown in Table 1.

### Peak oxygen consumption (V̇O2peak)

Regarding tissue volume, there were distinct positive correlations for V̇O_2peak_ and skeletal muscles in both sexes, with the highest correlations observed in the thighs. Positive correlations were also observed regarding heart volume; in males, there were strong correlations with lung volume.

**Fig. 2:**
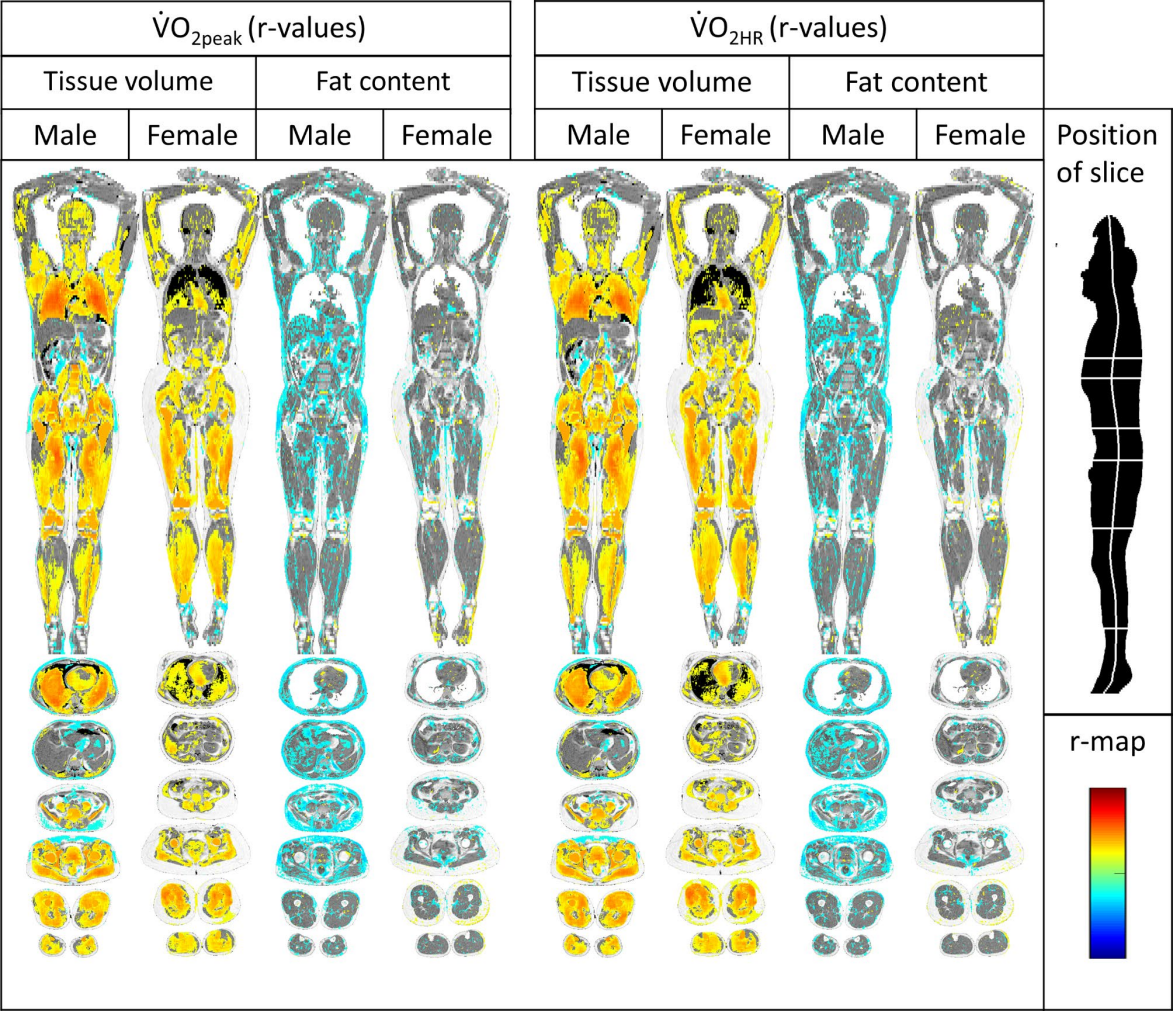
Imiomics maps of voxel-wise Pearson’s correlations (r-map). Significant (*p* < 0.05) voxel-wise correlation coefficients are shown and colour-coded by its value (r-value), see colour-scale in the lower left. From left to right: Tissue volume vs. V̇O2peak in males, tissue volume vs. V̇O2peak in females, fat content vs. V̇O2peak in males, fat content vs. V̇O2peak in females, tissue volume vs. V̇O2HR in males, tissue volume vs. V̇O2HR in females, fat content vs. V̇O2HR in males and fat content vs. V̇O2HR in females.

Weak correlations were found in males regarding fat fraction in subcutaneous fat. No discernible patterns regarding fat content were observed in females (Fig. 2).

### Oxygen pulse (V̇O2HR)

The same results that were observed for V̇O_2peak_ were identified in V̇O_2HR_.

### Maximal oxygen consumption scaled by body weight (V̇O2kg)

In males, strong negative correlations were seen between V̇O_2kg_ and tissue volume in subcutaneous fat, visceral fat and the liver. Strong positive correlations were also seen regarding lung volume in males. Females exhibited a similar pattern overall, but with more pronounced relationships in gluteal fat and less pronounced correlations with lung volume than those in males.

**Fig. 3:**
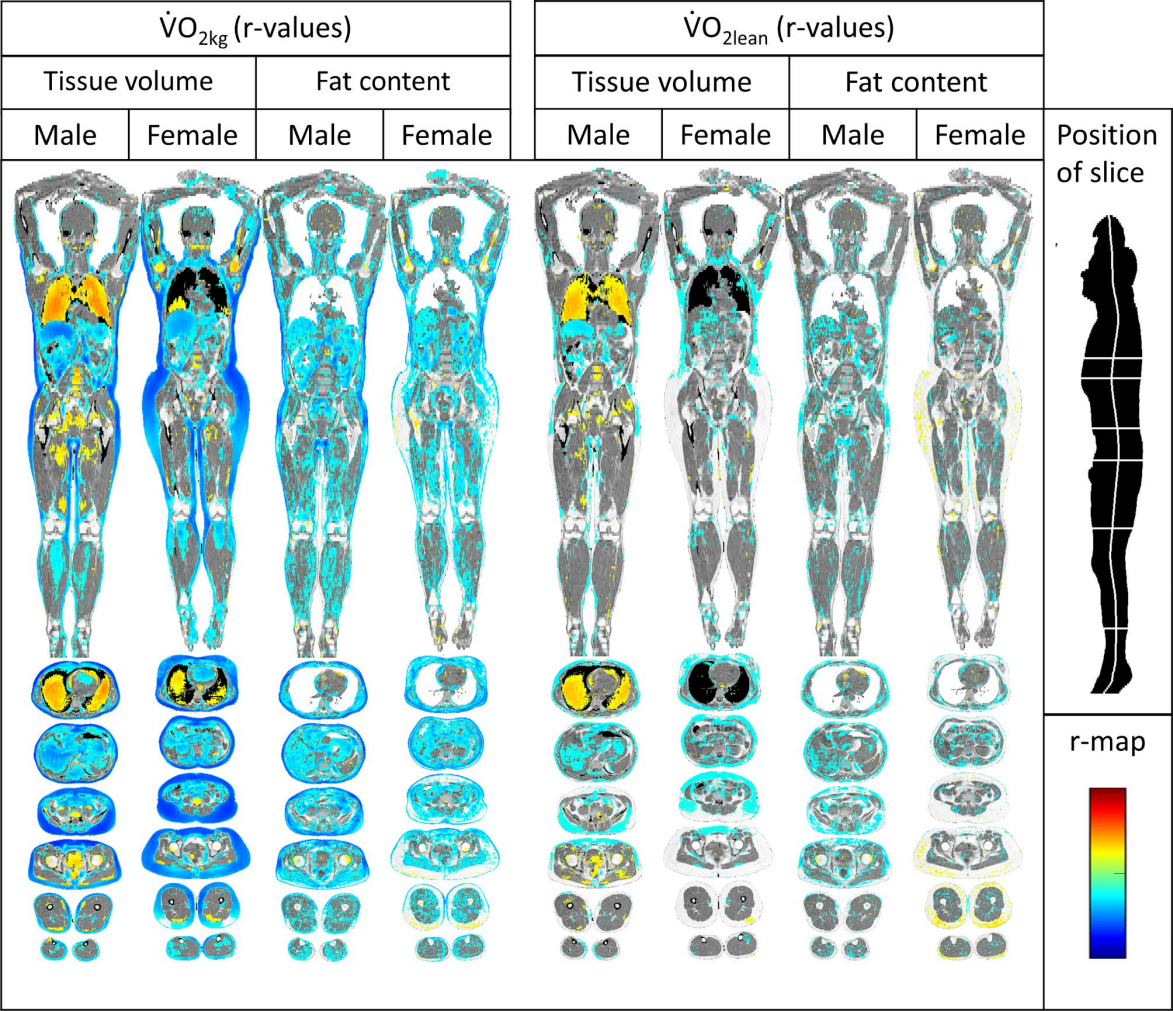
Imiomics maps of voxel-wise Pearson’s correlations (r-map). Significant (*p* < 0.05) voxel-wise correlation coefficients are shown and colour-coded by its value (r-value), see colour-scale in the lower left. From left to right: Tissue volume vs. V̇O2kg in males, tissue volume vs. V̇O2kg in females, fat content vs. V̇O2kg in males, fat content vs. V̇O2kg in females, tissue volume vs. V̇O2lean in males, tissue volume vs. V̇O2lean in females, fat content vs. V̇Olean in males and fat content vs. V̇O2lean in females.

In males, weak negative correlations were seen between V̇O_2kg_ and fat content in muscles, subcutaneous and visceral fat and the liver. In females, similar patterns were seen except for subcutaneous fat, for which a correlation was seen only in the trunk (Fig. 3).

### Maximal oxygen consumption scaled by lean mass (V̇O2lean)

In females, there were weak negative correlations between V̇O_2lean_ and tissue volume in subcutaneous fat in the trunk. In males, similar patterns were seen, with more pronounced correlations with liver size and distinct positive correlations with lung volume.

There were no distinct correlations between V̇O_2lean_ and fat content in either sex (Fig. 3).

### Respiratory exchange ratio at peak exercise (RERpeak)

In both sexes, there were weak negative correlations between RERpeak and tissue volume of skeletal musculature, subcutaneous fat in the trunk, visceral fat and liver. There was also a weak negative correlation with heart size in females.

**Fig. 4:**
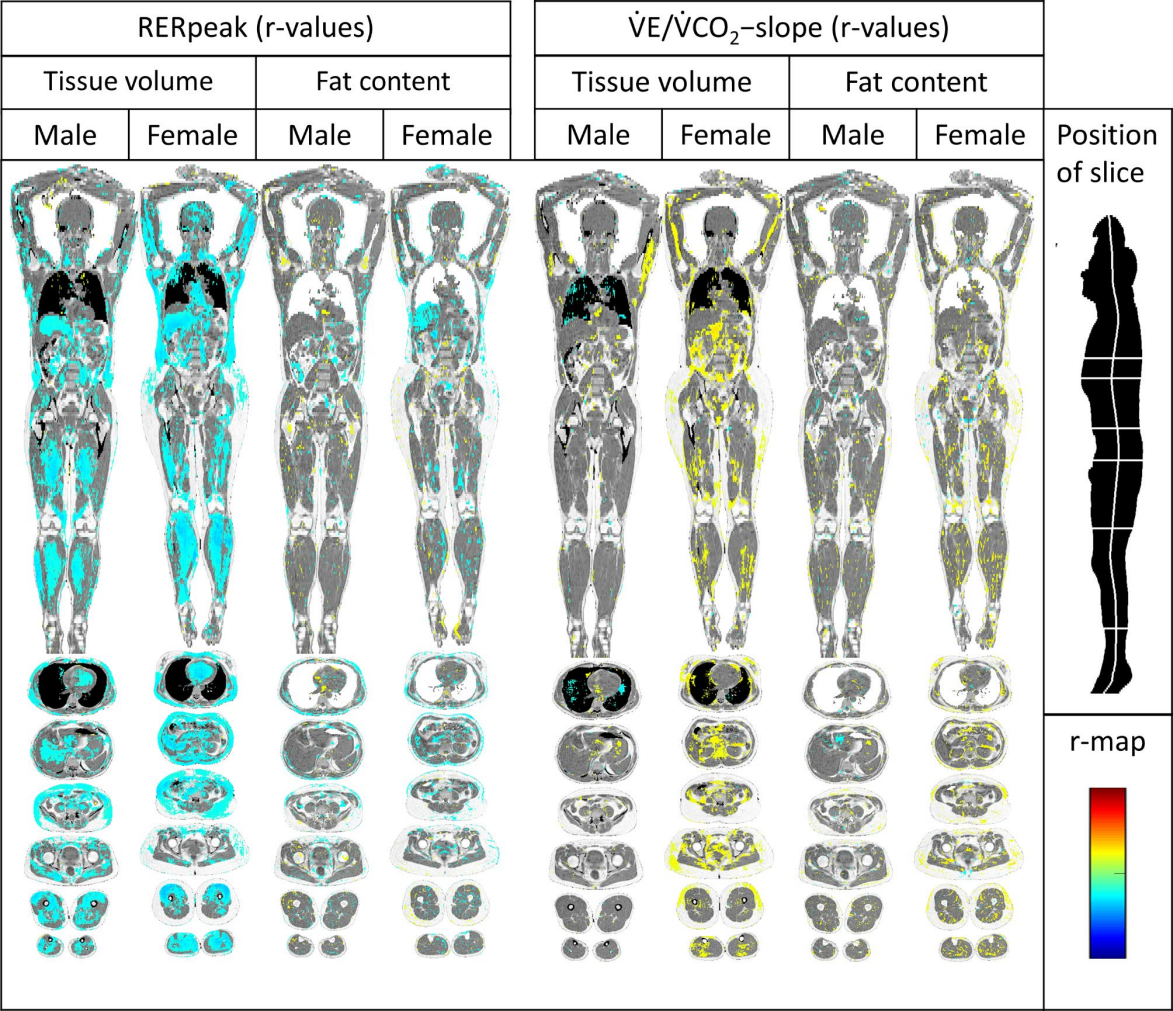
Imiomics maps of voxel-wise Pearson’s correlations (r-map). Significant (*p* < 0.05) voxel-wise correlation coefficients are shown and colour-coded by its value (r-value), see colour-scale in the lower left. From left to right: Tissue volume vs. RERpeak in males, tissue volume vs. RERpeak in females, fat content vs. RERpeak in males, fat content vs. RERpeak in females, tissue volume vs. V̇E/V̇CO2-slope in males, tissue volume vs. V̇E/V̇CO2-slope in females, fat content vs. V̇E/V̇CO2-slope in males and fat content vs. V̇E/V̇CO2-slope in females.

Regarding fat content, the only distinct patterns was in females, where there were negative correlations with liver and intramuscular fat content (Fig. 4).

### Ventilatory efficiency (V̇E/V̇CO2-slope)

In females, there were weak positive correlations between V̇E/V̇CO_2_ slope and tissue volume of visceral fat and humerus. In males, there were no clear correlations regarding tissue volume. There were no relationships between V̇E/V̇CO_2_ slope and fat content (Fig. 4).

### Work efficiency (ΔV̇O2/ΔWR)

In females, there were distinct positive correlations between **Δ**V̇O_2_/**Δ**WR and tissue volumes of subcutaneous fat in the thigh, gluteal and arm regions, and weak correlations in the liver and the heart. There were no such relationships in males.

**Fig. 5:**
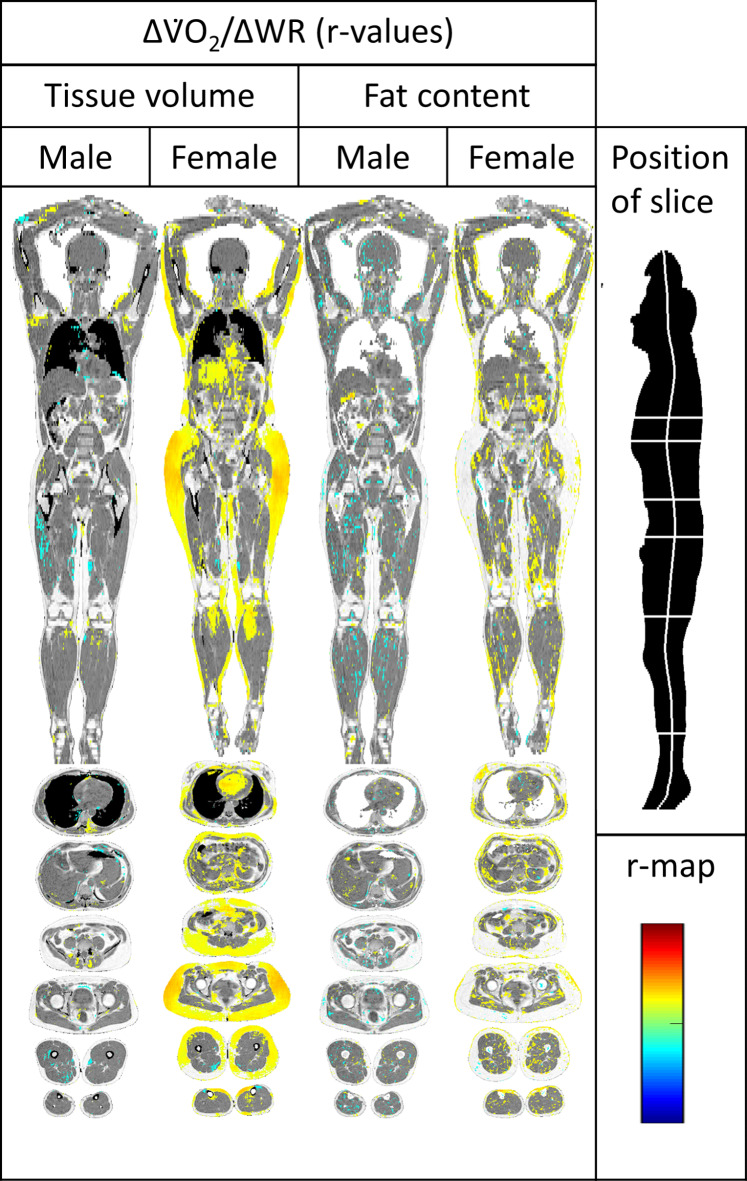
Imiomics maps of voxel-wise Pearson’s correlations (r-map). Significant (*p* < 0.05) voxel-wise correlation coefficients are shown and colour-coded by its value (r-value), see colour-scale in the lower left. From left to right: Tissue volume vs. **Δ**V̇O_2_/ΔWR in males, tissue volume vs. **Δ**V̇O_2_/ΔWR in females, fat content vs. **Δ**V̇O_2_/ΔWR in males, fat content vs. **Δ**V̇O_2_/ΔWR in females.

There were no convincing relationships between **Δ**V̇O_2_/**Δ**WR and fat content (Fig. 5).

## Discussion

In this study we examined the relationship between body composition variables by Imiomics MRI analysis and common indices from CPET.

The main findings were that higher V̇O_2peak_ was associated with larger volume of skeletal muscle mass, higher V̇O_2kg_ was strongly associated with lower tissue volume of subcutaneous fat, particularly gluteal fat and higher RERpeak was associated with lower volume of skeletal muscle, subcutaneous fat, visceral fat and the liver. Additionally, some associations appeared to differ between males and females; for example, higher V̇E/V̇CO_2_ slope was associated with more volume of visceral fat only in females, whereas larger lung volume was related to higher V̇O_2peak_, V̇O_2kg_ and V̇O_2lean_ only in males.

V̇O_2peak_ showed a distinct positive correlation with volume of skeletal muscles, finding in line with previous studies^29,30^. Potential reasons for this association could be: more O_2_ consuming tissue (muscle mass)^31^ greater blood volume (which increase the delivery of oxygen)^32^ and that individuals with larger muscles likely tend to engage more in exercise.

Regarding V̇O_2peak_ scaled by body weight, V̇O_2kg_, there was especially a strong negative correlation with tissue volume in subcutaneous fat. It is relatively well-established that individuals with higher fat mass or higher body weight have lower V̇O_2kg_. Hence, allometric scaling by bodyweight raised to a power (e.g., 2/3 or 2/4) has been suggested^21,33–35^. As fat mass is primarily metabolic inactive, it contributes only to the denominator (body-weight). Given these previous findings, the correlation with fat was expected. However, a more pronounced correlation with gluteal fat has not been established before. The underlying reason for this finding requires further investigation.

In contrast to V̇O_2kg,_ V̇O_2lean_ displayed weak correlation with tissue volume of subcutaneous fat in the trunk. Almost no correlation was seen with gluteal fat. This finding suggests that a greater volume of trunk fat – but not fat outside of the trunk, such as gluteal fat – is associated with lower cardiorespiratory fitness.

All the participants were 50 years old and therefore had the same expected maximum heart rate, consequently, the correlation patterns of V̇O_2peak_ and V̇O_2HR_ were found to be very similar. V̇O_2HR_ is commonly used as surrogate for stroke volume^1,2^; therefore, one could expect that higher V̇O_2HR_ would be associated with larger volume of the heart, this was also the case in our study.

RERpeak was negatively correlated with tissue volume of skeletal muscle, subcutaneous visceral fat and the liver in both sexes. There are previous studies indicating that RERpeak is lower in the obese^36,37^. This effect is likely due to lower effort levels, where larger individuals are not accustomed to exercise and may have more significant challenges in reaching maximal effort. However, an unknown underlying metabolic reason is also a possibility.

Interestingly, some correlations appeared to differ between the sexes. For example, V̇E/V̇CO_2_-slope was related to the tissue volume of visceral fat in females. Previous studies have indicated that V̇E/V̇CO_2_-slope is not correlated with body composition, or at least that obese individuals do not seem to have a higher V̇E/V̇CO_2_-slope^37,38^. Our results suggest that V̇E/V̇CO_2_-slope may be higher in females with more visceral fat. A potential mechanism behind this is that more visceral fat is linked to diastolic dysfunction^12,13^. There is also evidence that central obesity impairs lung function, particularly function residual capacity (FRC)^17^. However, it is hard to ascertain why there was no correlation between V̇E/V̇CO_2_-slope and visceral fat in males.

A distinct correlation pattern between **Δ**V̇O_2_/**Δ**WR and tissue volume, especially in subcutaneous fat was observed among females but not males. This contrast with previous studies which have reported a higher V̇O_2_ per work rate (higher intercept) but with the same slope (**Δ**V̇O_2_/**Δ**WR) in overweight subjects, assessed based on body mass index^1,33,39^.

Surprisingly, males exhibited a significant positive correlation between lung volume and V̇O_2peak_, V̇O_2kg_ and V̇O_2lean_. One can speculate that the volume of the lungs represents FRC, as the images were obtained at end expiration. To our knowledge, there is no previously known correlation between FRC and V̇O_2peak_. However, V̇O_2peak_ has been found to have a weak correlation with forced expiratory volume in 1 s without any differences between the sexes^40^.

### Strength and limitations

A strength of this study is the relatively large number of individuals who did both MRI with Imiomics and a CPET. As all participants were 50 years old, more studies with larger age range are needed to confirm our results are generalisable across different ages. We interpreted the Imiomics results visually, meaning that there was some degree of subjectivity on the part of the interpreter. It would been of interest to take into account the participants menopausal status; however, we did not have adequate data of this.

## Conclusion

The Imiomics method provided detailed insights about CPET indices association to tissue volume and fat content of different body structures. Our results suggest that there are variations in the correlation between body tissues and CPET indices depending on the tissues anatomical location; in addition, this appears to differ with regards to sex. Whereas some of the findings where considered expected and in line with previous research, others were more unanticipated and would be interesting objects for further examination (for example the association between lung volume and V̇O_2peak_ in males or the association between subcuteanous fat and **Δ**V̇O_2_/**Δ**WR in females).

Looking forward, besides the need to validate the findings, we see a potential for future studies aiming at discerning the underlying physiologic or metabolic mechanisms of our results. Furthermore, as several of the CPET indices, especially V̇O_2peak_, carries significant prognostic information, we consider it important also to map what associates with a beneficial CPET profile. This would allow to find new markers of health or test new targets of intervention and our study brings new knowledge to this area and could potentially spur new ideas or be a starting point for future research.

## Data Availability

Data is from the POEM study whose PI may be contacted at lars.lind@medsci.uu.se for requests. The ethics approval does not allow the authors to make all the data publicly available, by Swedish law.
